# Evolution of H9N2 avian influenza virus in embryonated chicken eggs with or without homologous vaccine antibodies

**DOI:** 10.1186/s12917-018-1391-6

**Published:** 2018-03-06

**Authors:** Haiyun Jin, Wan Wang, Xueqin Yang, Hailong Su, Jiawen Fan, Rui Zhu, Shifeng Wang, Huoying Shi, Xiufan Liu

**Affiliations:** 1 College of Veterinary Medicine, Yangzhou University, Yangzhou, Jiangsu 225009 People’s Republic of China; 2 Jiangsu Co-innovation Center for the Prevention and Control of Important Animal Infectious Diseases and Zoonoses, Yangzhou, 225009 China; 3 Department of Infectious Diseases and Pathology, College of Veterinary Medicine, University of Florida, Gainesville, FL 32611-0880 USA

**Keywords:** H9N2 influenza virus, Selective pressure of antibody, Mutation, Antigenic drift, Replication

## Abstract

**Background:**

Vaccines constitute a unique selective pressure, different from natural selection, drives the evolution of influenza virus. In this study, A/Chicken/Shanghai/F/1998 (H9N2) was continually passaged in specific pathogen-free embryonated chicken eggs with or without selective pressures from antibodies induced by homologous maternal antibodies. Genetic mutations, antigenic drift, replication, and pathogenicity of the passaged virus were evaluated.

**Results:**

Antigenic drift of the passaged viruses occurred in the 47th generation (vF47) under selective pressure on antibodies and in the 52nd generation (nF52) without selective pressure from antibodies. Seven mutations were observed in the vF47 virus, with three in PB2 and four in HA, whereas 12 mutations occurred in the nF52 virus, with three in PB2, two in PB1, four in HA, one in NP, one in NA, and one in NS. Remarkably, the sequences of the HA segment from vF47 were 100% homologous with those of the nF52 virus. Both the vF47 and nF52 viruses showed enhanced replication compared to the parental virus F/98, but higher levels of replication and pathogenicity were displayed by nF52 than by vF47. An inactive vaccine derived from the parental virus F/98 did not confer protection against challenges by either the vF47 or nF52 virus, but inactive vaccines derived from the vF47 or nF52 virus were able to provide protection against a challenge using F/98.

**Conclusion:**

Taken together, the passage of H9N2 viruses with or without selective pressure of the antibodies induced by homologous maternal antibodies showed genetic variation, enhanced replication, and variant antigenicity. Selective pressure of the antibody does not seem to play a key role in antigenic drift in the egg model but may impact the genetic variation and replication ability of H9N2 viruses. These results improve understanding of the evolution of the H9N2 influenza virus and may aid in selecting appropriate vaccine seeds.

## Background

The H9N2 influenza virus has spread rapidly in chickens throughout Asia since the 1990s [1, 2]. This virus, which was initially isolated from chickens in 1994, has caused severe economic losses for the poultry industry in China [2–6]. To control the spread of H9N2 avian influenza, a vaccination program for the H9N2 influenza virus has been widely implemented throughout mainland China over the past two decades [7, 8]. While this immunization program effectively reduced the economic loss caused by H9N2 influenza virus in chickens, it did not prevent the spread of H9N2 avian influenza through much of China [8]. In many Chinese provinces, H9N2 avian influenza now not only circulates in chickens but has also spread to pigs [9, 10] and wild and domestic birds [6, 11].

There are two scenarios under which the transmission of H9N2 avian influenza virus occurs. The first is a sporadic outbreak of H9N2 avian influenza virus in chicken farms occurring despite chicken vaccination, and can even occur in animals with a high antibody titer against H9N2 influenza [7, 8]. This scenario implies that the existing vaccine may not provide complete protection against infection by the prevalent H9N2 virus. Thus, vaccination against H9N2 influenza virus will not prevent the shedding of a common strain of H9N2 and may play a role in driving the evolution and spread of H9N2 influenza virus. The second scenario under which H9N2 avian influenza virus is transmitted relies on the ability of the H9N2 virus to reassort with other influenza virus subtypes to generate new influenza viruses. In this manner, an H9N2 virus supplied internal genes to an H5N1 virus in 1997 [2, 12, 13] and to H7N9 [14, 15] and H10N8 avian influenza viruses in 2013 [16]. Pu et al. [17] reported genotype 57 of the H9N2 chicken virus as the fittest virus to emerge over the 10 years of co-circulation of multiple H9N2 genotypes, that it had changed antigenicity and improved adaptability in chickens, and finally provided all of their internal genes to the novel H7N9 viruses [17]. The new virus genotype 57 displayed a strong advantage in its ability to escape the selective pressure of hosts.

The immune selective pressure of host drives influenza virus evolution [18]. During this process, antigenic drift can allow an influenza virus to escape recognition by virus-neutralizing antibodies [18, 19]. To date, all reported antigenic variations of H9N2 influenza virus were characterized based on their reactivity with monoclonal antibodies in a cell model [20, 21]. However, the effect of vaccine-induced polyclonal antibodies on the evolution of H9N2 influenza virus has not been quantitatively explored in either embryonated chicken eggs or a chicken model. Overall, chickens are suitable hosts for influenza virus, possess a whole functioning immune system, and can be a good model for studying the influence of vaccine-induced antibody on the evolution of H9N2 avian influenza virus. Moreover, specific pathogen-free (SPF) embryonated chicken eggs have an immature immune system compared with mature chickens. Even though eggs are not natural hosts of avian influenza, they can be a useful artificial model to evaluate the effects of selective pressures from antibodies (SPA) induced by homologous maternal antibodies on the evolution of H9N2 influenza viruses. Given that the H9N2 virus is still widely spreading and vaccination is the only current effective means with which to control H9N2 influenza, it would be useful to evaluate the influence of vaccine-induced antibodies on the evolution of the H9N2 avian influenza virus.

Sun et al. reported that, in China, the prevalent H9N2 avian influenza virus prior to 2000 was the BJ/94-lineage, and after 2004 the F/98-lineage became dominant. Currently, the F/98-lineage is still the prevalent genotype in China [22, 23]. In this study, A/Chicken/Shanghai/F/98 (H9N2, F/98) avian influenza virus, which is used in most regions of China as an early vaccine seed for H9N2 avian influenza [8, 21], was serially passaged in SPF embryonated chicken eggs with or without SPA induced by homologous maternal antibodies. The resulting passaged viruses were analyzed to investigate the effects of selective pressure of the antibody on the antigenic drift, genetic evolution, replication, pathogenicity, and immunogenicity of these viruses.

## Methods

### Animals

Ten-day-old of incubation SPF embryonated chicken eggs, SPF hens and cocks were purchased from the Shandong Poultry and Breeding Farms, China, and housed under SPF conditions. All animal experiments were approved by the Jiangsu Administrative Committee for Laboratory Animals (permission number SYXK-SU-2007-0005) and complied with the Jiangsu Laboratory Animal Welfare and Ethics guidelines of the Jiangsu Administrative Committee of Laboratory Animals [24].

### H9N2 virus, MDCK cells and vaccine preparation

A/Chicken/Shanghai/F/98 (H9N2, F/98) influenza virus was isolated in Shanghai in 1998, purified and stocked by the Animal Infectious Disease Laboratory, School of Veterinary Medicine, and Yangzhou University [25]. The H9N2 avian influenza virus F/98 was propagated in the allantoic cavity of 10-day-old SPF embryonated eggs. The virus preparation was stored at − 70 °C until use. All experiments involving live viruses and animals were carried out in biosafety level (BSL) 2+ containment, as approved by Yangzhou University.

Madin-Darby canine kidney (MDCK) cells were grown in DMEM media (Invitrogen) containing 10% fetal bovine serum (FBS) (Invitrogen), and cultured in an incubator with 5% CO_2_, 37 °C.

The F/98 strain was inactive by adding 0.2% formalin (*v*/v) for 24 h at 37 °C. Inactivation was confirmed by the absence of detectable infectivity after two blind passages of formalin-treated allantoic fluid in embryonated eggs. The inactive allantoic fluid was emulsified in two parts of paraffin oil (v/v), which is currently used commercially as an adjuvant for veterinary vaccine production [26].

Hemagglutination (HA) and hemagglutination inhibition (HI) assays, the antisera preparation of F/98 virus, routine HA and HI tests were used as outlined in the OIE *Manual of Diagnostic Tests and Vaccines for Terrestrial Animals* (http://www.oie.int/manual-of-diagnostic-tests-and-vaccines-for-terrestrial-animals/).

Three, four-week-old SPF chickens were immunized by intramuscular injection of 0.3 ml oil-emulsion of inactive whole virus vaccine of the indicated viruses. Sera were taken in chickens at 3 weeks after immunization and treated with receptor-destroying enzyme (RDE, Cholera filtrate, Sigma, USA) to remove non-specific haemagglutination inhibitors, as above described an HI test was performed. Samples were regarded positive for inhibition of haemagglutination at a dilution ≥1:16.

### HI test for yolk antibody

Three ten-day-old of incubation SPF embryonated chicken eggs either directly from SPF hen, or from the vaccinated SPF hens inseminated with SPF cocks. For yolk immunoglobulin extraction using a simplified chloroform polyethylene-glycol procedure, 2 ml of egg yolk was mixed with an equal volume of phosphate-buffered saline, and then added to 4 ml of chloroform. After mixing well, the yolk was centrifuged at 3500 rpm for 10 min. The supernatant was collected and used for antibody tests. The HI test was performed according to the standard procedures recommended by the OIE *Manual of Diagnostic Tests and Vaccines for Terrestrial Animals*. Briefly, the yolk samples were treated with a receptor-destroying enzyme (RDE) (Denka Seiken Co. Ltd., Tokyo, Japan) at 37 °C for 20 h to eliminate non-specific inhibitors of hemagglutination. HI titers obtained from a purified yolk and RDE mixture (25 ml egg yolk + 75 ml RDE provided a starting dilution of 1:1) were defined as the reciprocal of the highest dilution of yolk, which completely inhibited hemagglutination of 4 hemagglutination units of the virus with a 1% solution of chicken red blood cells. Samples with HI titers under 16 were considered negative.

### F/98 (H9N2) serial passaged in the SPF embryonated chicken eggs with or without SPA

**Fig. 1:**
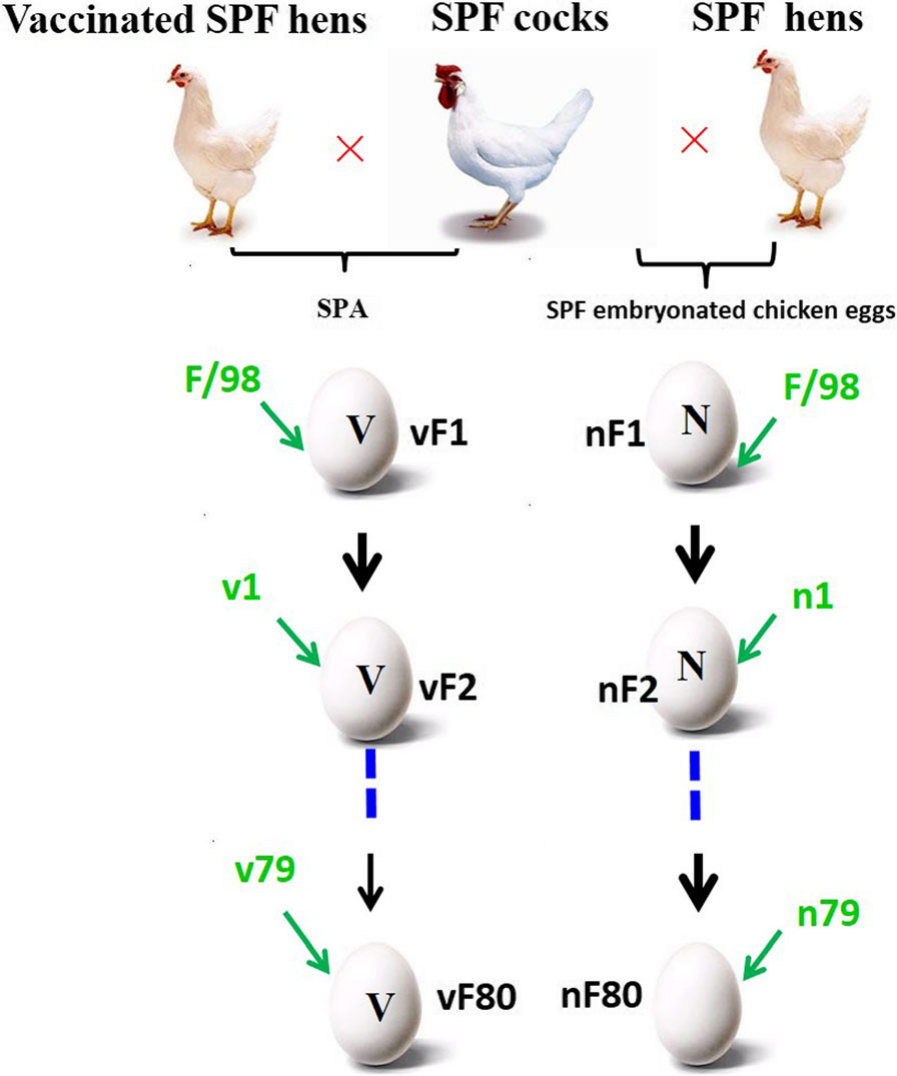
Virus passage process. These images in Fig. 1 were made by authors and permitted to use

Three SPF hens were vaccinated three times with oil emulsion vaccine of F/98 strain. When HI titer in the serum diagram for the experiment design of the vaccinated hens was up to 512 [27], the vaccinated SPF hens were artificially inseminated with SPF cocks, resulting in the eggs with 16 or 32 HI titers of the yolk antibody against the parental virus F/98. These eggs were used as the SPA model. Meanwhile, the SPF embryonated chicken eggs directly bought from the same chicken farm served as a control without SPA. Three eggs from either SPA or the SPF embryonated chicken eggs were inoculated with 10^6^ the 50% egg infectious dose (EID_50_) of the F/98 virus. The allantoic fluid of each generation pooled from 3 eggs with or without SPA was collected after 72 h incubation. Part of allantoic fluid was saved at the − 70 °C, part of allantoic fluid were combined and used for the next passage in another three eggs with or the SPF embryonated chicken eggs, respectively, until 80 passages (Fig. 1). The embryo means death time (MDT) of the indicated passaged virus were calculated as described by Eugster AK. Et al [28], and EID_50_ titers of the indicated passaged virus were determined by serial titration of viruses in SPF embryonated chicken eggs using the Reed and Muench method [29].

### Assay of viral antigenic drift

Haemagglutination antibody levels of each passaged virus against F/98 virus were determined by a haemagglutination inhibition (HI) assay. When HI titers of the passaged virus were up to 8-fold difference from that of the parental virus F/98, antigenicity of this passaged virus was believed to be drift [20]. These passaged virus presented in the allantoic fluid were divided two parts. The genes of one part virus were directly deep sequencing, another part virus was plaque purified twice in MDCK cells. Ten clones of each indicated virus were picked up, and passaged once in the allantoic cavities of 10-day-old chicken eggs for 72 h at 37 °C, and did Sanger sequencing for whole genome sequences [30].

### Deep sequencing

Viral RNA was extracted from allantoic fluid of the indicated passaged viruses using the High Pure RNA isolation kit (Roche). RNA was subjected to reverse transcription-PCR (RT-PCR) using 18 primer sets that cover the entire viral genome. These primer sets were designed according to the genome sequences of the F/98 virus and by using Primer Premier 5.0 software. The fragments, approximately 600 to 800 nucleotides in length, were sequenced using the Illumina HiSeq2000 sequencing platform in the Chinese National Human Genome Center, Shanghai. Briefly, a library was constructed with TruSeq DNA sample prep kit set A. The DNA library was diluted and hybridized to the paired-end sequencing flow cells. DNA clusters were generated on a cBot cluster generation system with the TruSeq PE cluster generation kit v2, followed by sequencing on a HiSeq 2000 system with the TruSeq SBS kit v2. The threshold for the detection of single-nucleotide polymorphisms (SNP) was set manually at 10% of the population.

### Sanger sequencing

Genome RNAs of viral clones were extracted from culture supernatants using the QIAamp viral RNA kit (Qiagen). Genes were reverse transcribed and amplified using a OneStep RT-PCR kit (Qiagen). Primers were the same as those used in the deep sequencing assay. The amplified cDNA products were excised from agarose gels and purified using a QIAquick gel extraction kit (Qiagen). Full-genome DNAs were Sanger sequenced by TAKARA BIOTECHNOLOGY (DALIAN) CO., LTD., and the sequence data were analyzed using GenScan software.

The positions of amino acid substitutions on the HA molecule were analyzed on the 3-dimensional structure obtained from the Protein Databank (PDB accession number, 1JSD) with the RasMol 2.7.3 program.

### Replication and transmission in chickens

For studying the viral replication and pathogenicity in chicken, groups of six 4-week-old SPF chickens were oral, intranasally, or intratracheally inoculated with 0.2 ml of 10^6^ EID_50_ of the parental virus F/98, the passaged viruses or phosphate buffer solution (PBS) control. Tissue samples (trachea and lung) from the inoculated chickens were collected at 3 days and 5 days post-inoculation (dpi) (28, 40). Briefly, three SPF chickens were euthanatized using CO_2_ asphyxiation at designated times and half of the tissues were harvested, washed and ground into 20% (*w*/*v*) suspension in 1 ml PBS. Virus titers in the trachea and lung were determined in 10-day-old SPF embryonated chicken eggs accordingly (43). Another half part of the tissues were fixed with 10% neutral buffered formalin, embedded in paraffin, sectioned at 5 mm and routinely processed for staining with hematoxylin and eosin for histopathological examination (47). These experiments were repeated three times.

For studying the viral transmission, twelve 4-week-old SPF chickens for each virus were divided into: (i) inoculated group (three chickens), (ii) direct contact group (three chickens), (iii) airborne contact group (three chickens), (iv) PBS control group (three chickens) (28, 40). The infected group was inoculated oral, intranasally and intratracheally with 10^6^ EID_50_ of indicated virus. The airborne contact group was placed in a cage directly adjacent to the infected group with a distance of 50 cm between cages. At day 3, 5, 7 and 9 post-inoculation, tracheal and cloacal swabs from chickens were collected in 1 ml of PBS containing antibiotics, following freeze/thaws once, and were centrifuged at 3000 rpm for 10 min. Of the resulting supernatant, 0.2 ml were taken and the EID_50_ titers of tracheal and cloacal swabs collected from the indicated passaged virus were determined by serial titration of viruses in SPF embryonated chicken eggs using the Reed and Muench method [29]. Sera collected from each experiment bird at 10 and 20 dpi were tested by HI assay to check the antibody in the serum of chickens against the indicated viruses [31]. The experiment was repeated three times.

### Antigenic variant of the passaged viruses in chickens

To study the cross - immunogenicity of the passaged virus, 135 4 - week - old SPF chickens were divided into 5 groups, A, B, C, D and E (Table 3). In group A, each subgroup of 9 chickens were immunized with PBS and challenged with PBS and the indicated 0.3 ml inactivated oil-emulsion virus [32], respectively; in group B, each subgroup of 9 chickens were immunized with the vaccine of F/98 virus, and challenged with the indicated virus, respectively; In group C, or D, or E, each subgroup of 9 chickens were immunized with the indicated passaged virus, and challenged with the indicated virus, respectively (Table 3). On days 3 and 5 post-challenge, the viruses from challenged chickens were isolated from trachea and cloaca. The experiment was repeated three times.

### Statistics

Data were presented as the geometric means and standard deviations for all assays. The Mann-Whitney U test (GraphPad Software, Inc., San Diego, CA) was used to evaluate the replication ability of strains and the tissue lesion in chickens challenged with different viruses. A *P* value of 0.05 was considered statistically significant.

## Results

### Adaption of H9N2 avian influenza virus F/98 passaged in SPF eggs with or without SPA

To explore the role of SPA on the evolution of H9N2 avian influenza virus, the F/98 virus was serially passaged in SPF embryonated eggs in the presence or absence of SPA, and the allantoic fluid of each generation was collected and stocked (Fig. 1). HI titers of the passaged virus in these fluids were determined. Compared to the parental virus F/98, the HI titers of the passaged viruses were significantly decreased to 32-fold starting with vF47 (vF47: and nF52. In contrast, the HI titer of nF47 was only two-fold lower than that of F/98, indicating that antigenic drift had occurred in the passaged viruses vF47 and nF52 (Table 1). This result indicates that the antigenicity of F/98 H9N2 avian virus changed after being continually passaged either with or without SPA.

**Table 1:** Adaptation of H9N2 avian influenza virus in SPF embryonated chicken eggs with or without SPA

Characters	F/98	nF47	nF52	vF47
HI titer	512	256	16	16
Change in HI titer compare to F/98		2-fold	32-fold	32-fold
EMDT (h)^a^	86	79	68.5	75
Change in EMDT(h)compare to F/98		7	17.5	11
EID_50_ (log10 EID_50_/0.2 ml)	6.67	7.64	8.74	7.77
Changed in EID_50_ compare to F/98 (log10 decrease)		9.3-fold**	117.5-fold**^##^	12.5 fold**
Mutations^b^ compare to F/98 (start-end passage of mutant)		PB2: S155 N, C228Y, K375R (nF46-nF80)	PB2:S155 N, C228Y, K375R (nF46-nF80)	PB2: I185K, C228Y, K375R (vF43-vF80)
		PB1: none	PB1:T156A, R189K (nF49-nF80)	PB1: none
		PA: none	PA: none	PA: none
		HA: K131R (nF30)	HA: K131R(nF30-nF80), S145 N, G181E, A198V (nF52-nF80)	HA: K131R, S145 N, G181E, A198V (vF47-vF80)
		NP: none	NP: R99K (nF50-nF80)	NP: none
		NA: none	NA: A459T (nF52-nF80)	NA: none
		M: none	M: none	M: none
		NS1: none	NS1: A143T (nF50-nF80)	NS1: none

^a^EMDT: Embryo mean death time

^b^each gene represent results from 10 clones

**P < 0.01 for the 50% egg infectious dose of nF47, vF47, and nF52 was significantly higher than that of their parental virus F/98

^##^*P* < 0.01 for the 50% egg infectious dose of nF52 was significantly higher than that of nF47 and vF47

The passaged viruses vF47, nF47 and nF52 in the allantoic fluid were further deeply sequenced. We did not determine any viral quasispecies. Simultaneously, the passaged viruses vF47, vF60, vF70, vF80, nF47, nF52, nF60, nF70, and nF80 in the allantoic fluid were twice purified by plaque purification in MDCK cells (10 plaques of each indicated virus were picked up and sequenced to determine the purity of the passage viruses). Those viruses were then passaged once in the allantoic cavities of 10-day-old chicken eggs. A sequence analysis of these viruses confirmed that serial passage in allantoic cavities of the egg model did not lead to the formation of viral quasispecies.

The F/98, nF47, vF47, and nF52 viruses passaged in the allantoic fluid of eggs were selected for further research. We found that the embryo means death time (EMDT) of nF47, vF47, and nF52 in SPF embryonated chicken eggs was reduced by 7 h, 11 h, and 17.5 h, respectively, compared to the 86 h EMDT of the parental F/98 virus. The 50% egg infectious dose (EID_50_) of nF47, vF47, and nF52 was significantly higher than that of their parental virus F/98 by 9.3-fold, 12.5-fold, and 117.5-fold, respectively (Table 1, *p* < 0.01). Notably, vF47 and nF47 shared similar EID_50_ titers. However, after five more passages, the EID_50_ of the nF52 virus was significantly increased compared to nF47 and vF47 (Table 1, *p* < 0.01).

### SPA affects the genetic mutations of H9N2 avian virus

According to HI titers of the passaged viruses, vF47 and nF52 viruses were the first generations that displayed antigenic change compared to the parental virus F/98. To identify the effect of SPA on the genetic mutations of the H9N2 avian virus, the genomes of vF47, vF60, vF70, vF80, nF47, nF52, nF60, nF70, and nF80 virus passaged in the allantoic fluid of eggs were sequenced by Sanger sequencing. Compared to the parent virus F/98, nF47 has four amino acid substitutions, three in the PB2 gene (S155 N, C228Y, K375R) and one in the HA gene (K131R). Conversely, vF47 has seven amino acid mutations, three in the PB2 gene (I185K, C228Y, K375R) and four in the HA gene (K131R, S145 N, G181E, A198V). Additionally, nF52 has 12 amino acid mutations, three in the PB2 gene (S155 N, C228Y, K375R), two in the PB1 gene (T156A, R189K), four in the HA gene (K131R, S145 N, G181E, A198V), one in the NP gene (R99K), one in the NA gene (A459T), and one in the NS1 gene (A143T) (Table 1). The genome sequences of vF60, vF70, and vF80 were each the same as that of vF47. The nF60, nF70, and nF80 genome sequences were each the same as that of nF52. The sequencing results show that vF47 has three more mutations in the HA gene (S145 N, G181E, A198V) and one different mutation in the PB2 gene (S155 N) compared with nF47. However, after five more passages, additional mutations distributed in the NA, PB1, NP, and NS1 genes of the nF52 virus were observed. Interestingly, vF47 and nF52 shared the same mutations in the HA gene, even though vF47 and nF52 resulted from the different eggs with and without SPA (Table 1).

To determine whether the amino acid changes observed in vF47 or nF52 occur in H9N2 wild-type isolates, sequences from the National Institute of Allergy and Infectious Diseases database (http://www.fludb.org) between 1994 and 2015 (from 1 January 1994 to 20 December 2015) were analyzed. Most H9N2 wild-type isolates shared common mutations with vF47 or nF52. Over the past 20 years, the mutations C228Y and K375R of the PB2 gene that we observed in the passaged viruses displayed high mutation frequencies of 92–100% in wild-type viruses. Additionally, the mutation frequencies of K131R, S145 N, G181E, and A198V in the HA gene that we observed in the vF47 and nF52 viruses were 96.3–99.4%, 3.7–18.1%, 0–72%, and 30.2–42.4% in wild-type viruses, respectively. In comparison, the A143T mutation in the NS1 gene that we observed in the nF52 virus had a mutation frequency of 18.8–43.2% in wild-type viruses. Notably, the mutations S145 N and G181E in HA were significantly increased in wild-type isolates, with 18.1% and 54% over recent years.

### Mutants mapping of the H9 HA molecule

**Fig. 2:**
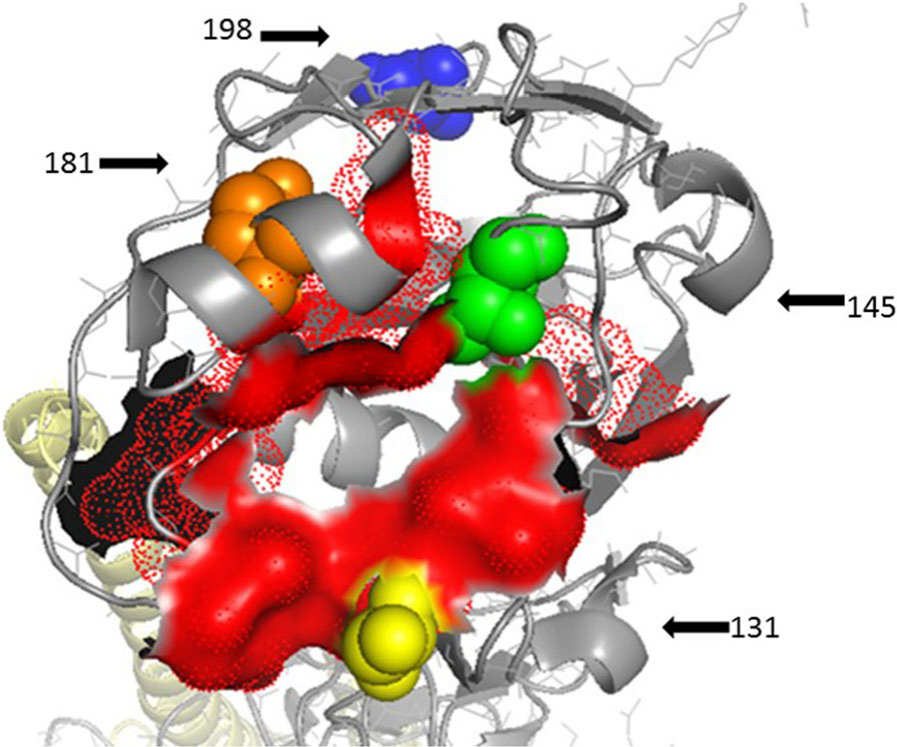
Mutants mapping of HA. Schematic representation of monomer structures of the H9 haemagglutinin molecule showing the locations of amino acid substitutions on HA1. Amino acid changes of escape mutants selected with or without homologous vaccine antibody against Ck/SH/F/98 (H9N2). The positions of the mutations sites are shown as yellow for 131 site, green for 145 site, apricot yellow for 181 site, and blue for 198 site. Red for receptor binding pocket of HA1 in H9 virus. Amino acid positions correspond to H9 numbering. Images were created with RasMol 2.7.3

As described above, vF47 and nF52 shared the same mutations carrying K131R (yellow), S145 N (green), G181E (apricot yellow), and A198V (blue) in the HA gene. The locations of the amino acid substitution in the HA of H9 are illustrated on the three-dimensional map of F/98 HA (Fig. 2). The results show that all mutants distributed close to the hemagglutinin receptor-binding site (red). Interestingly, substitution of S145 N provided one more potential glycosylation site in both the vF47 and nF52 viruses.

### H9N2 viruses passaged with or without SPA display different replication and pathogenicity in chickens

**Fig. 3:**
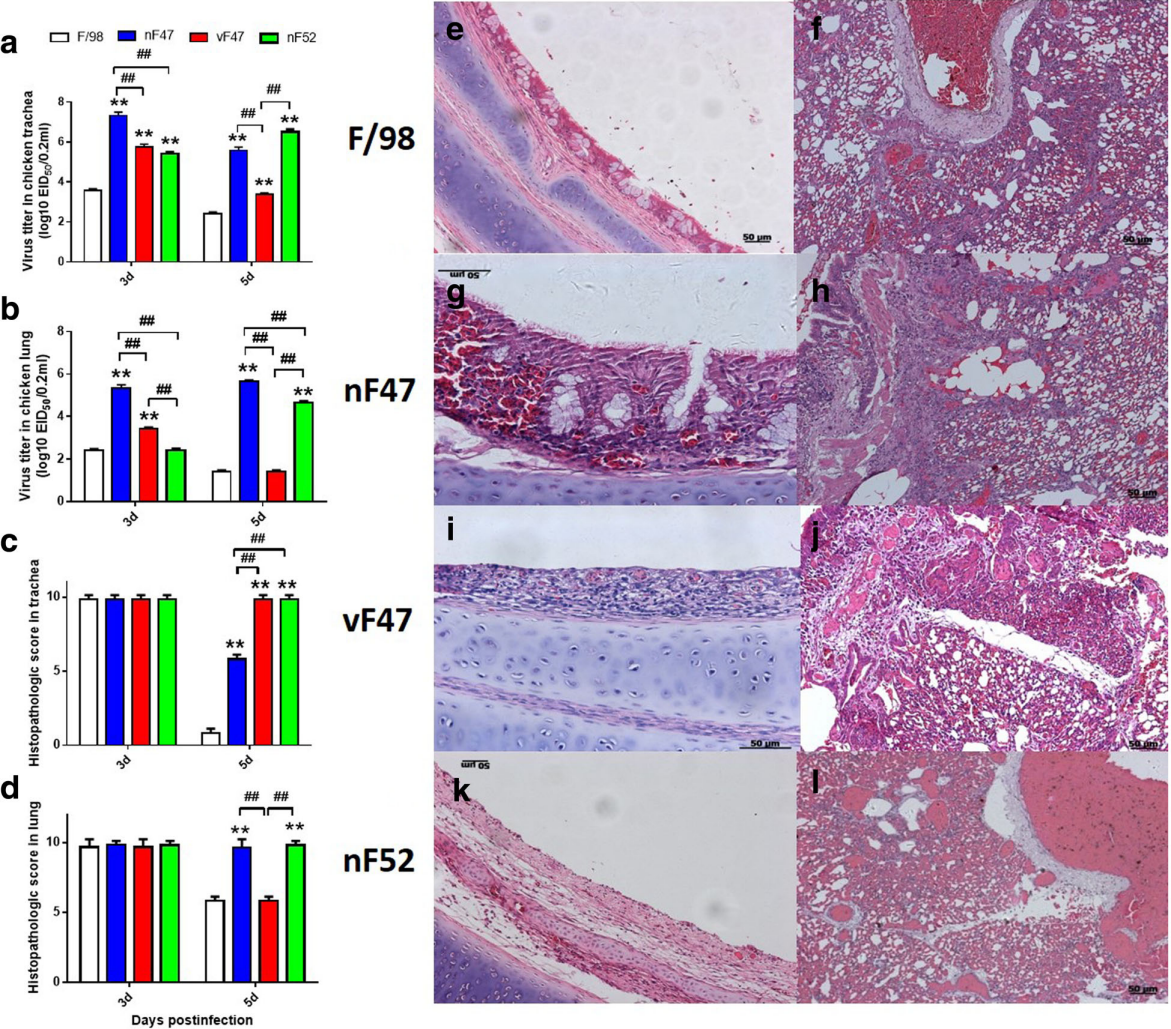
Replication and pathological changes of the viruses in chickens. Groups of three chickens were inoculated orally, intranasally, and intratracheally with 10^6^ EID_50_ of F/98 or the passaged viruses. **a**-**b** Replication ability of the viruses in trachea and lung of chickens were evaluated at 3 and 5 dpi. For virus titration in SPF eggs. The average of each group is shown, with error bars representing the SD. **, *P* < 0.01 compared with the value for the parental virus F/98 group; ##, *P* < 0.01 for the passaged viruses group compared each other. Data are representative of three independent experiments. **c**-**d** Histology scores of viruses in the trachea (**c**) and lung (**d**) chickens at 3 and 5 days after inoculated with H9N2 virus. Horizontal lines show the mean, error bars represent the SD, and data are representative of three independent experiments. **e**-**l** Representative H&E histology sections of the viruses at 5 d after inoculated with different virus. **e**-**f** F/98 virus-infected trachea and lung; **g**-**h** nF47 virus-infected trachea and lung; **i**-**j** vF47 virus-infected trachea and lung; **k**-**l** nF52 virus-infected trachea and lung. The histopathological changes were scored as follows. For trachea, 0: normal; 1: congestion; 2: cilia loss; 3: a few inflammatory cell infiltration; 7: a lot of inflammatory cell infiltration; for lung, 0: normal; 1: congestion; 2: hemorrhage; 3: inflammatory cell infiltration in bronchial submucosa; 7: a lot of inflammatory cell infiltration in bronchial submucosa and alveolus

We further examined the replication and pathogenicity of the viruses passaged with or without SPA by comparing the virus titers and pathology parameters in chickens. The results show that the viral load of the passaged viruses in the trachea were 100–10,000-fold higher than that of their parental virus F/98 (Fig. 3a, *p* < 0.01) at 3 and 5 days post-infection (dpi). The titers of vF47 in the trachea were significantly lower than those of nF47 at 3 and 5 dpi (Fig. 3a, *p* < 0.01), and they were also lower than that of nF52 at 5 dpi (Fig. 3a, *p* < 0.01). Additionally, the titers of nF47 and nF52 at 5 dpi were not only higher than their corresponding titers at 3 dpi (Fig. 3b, *p* < 0.01), but they were also higher than those of their parental virus F/98 and of vF47 (Fig. 3b, *p* < 0.01). The titer of vF47 decreased in the lung (Fig. 3b, *p* < 0.01) to a level similar to that of the parental virus F/98.

The histopathological lesions of the trachea and lung in chickens infected with one of the passaged viruses, or with the wildtype virus F/98, had similar levels of severity at 3 dpi (Fig. 3c - k). At 5 dpi, the passaged virus vF47 displayed an increased level of pathogenicity only in the trachea (Fig. 3c, Fig. 3i, *p* < 0.01), whereas the passaged viruses nF47 and nF52 showed enhanced pathogenicity levels in both the trachea and lung (Fig. 3c - d, *p* < 0.01; Fig. 3g - h, k- l) compared with those of the parental virus F/98. Additionally, nF47 and nF52 each showed a higher pathological score than that of vF47 at 5 dpi (Fig. 3d, *p* < 0.01).

### SPA improves the airborne transmission characteristics of H9N2 influenza virus

We set up transmission experiments with the passaged viruses nF47, vF47, nF52 and their parental virus F/98 to further explore the effect of SPA on the transmission route of the H9N2 influenza virus [31]. The results show that all tested viruses could spread by direct contact. In the directly inoculated group, the shedding of vF47 was detected at 3 and 5 dpi in the trachea of chickens and was measured at 5, 7 and 9 dpi in the cloaca of chickens. This is the same shedding pattern observed for the parental virus F/98. In contrast, the nF47 virus could be isolated at 3, 5, 7, and 9 dpi, and the nF52 virus was isolated at 3, 5, and 7 dpi in the trachea of chickens, thus indicating that nF47 and nF52 each displayed a longer shedding period than vF47 and F/98 did in the trachea of chickens. In the airborne contact group, the F/98, nF47, and vF47 viruses could spread by airborne transmission, whereas nF52 lacked the ability to undergo airborne transmission (Table 2). These features were further confirmed by the result of the serologic analysis of HI assays, in which serum collected from the airborne contact chickens that were inoculated with nF52 showed negative reactivity against the nF52 virus, whereas sera from the airborne contact chickens that were inoculated with F/98, nF47, or vF47 viruses displayed positive reactivity against the indicated viruses at 10 and 20 dpi (Table 2). This finding implies that the presence of SPA during serial passaging might be beneficial for preserving the ability of H9N2 influenza virus to spread via airborne transmission as well as for maintaining the same shedding pattern as the parental virus F/98.

**Table 2:** Isolation for H9N2 viruses of tracheal and cloacal swabs in chickens in transmission experiment

Virus	Ways of infection	Tracheal swabs (virus titers^b^)				Cloacal swabs (virus titers^b^)				Mean HI titers of chickens (2^n^)	
		3 d	5 d	7 d	9 d	3 d	5 d	7 d	9 d	10 d	20 d
F/98	Directly inoculation^a^	9/9(3.3 ± 0.2)	6/9(1.85 ± 0.2)	0/9	0/9	0/9	2/9(1.65 ± 0.1)	6/9(1.8 ± 0.4)	3/9(<1^c^)	5 ± 0.7	9 ± 0.5
	Airborne contact^a^	2/9(1.7 ± 0.1)	4/9(<1^c^)	2/9(<1^c^)	0/9	0/9	4/9(1.1 ± 0.1)	2/9(<1^c^)	0/9	3 ± 1.9	9 ± 0.3
nF47	Directly inoculation^a^	9/9(5.3 ± 0.4)	9/9(3.45 ± 0.3)	9/9(1.65 ± 0.2)	6/9(<1^c^)	0/9	4/9(2.4 ± 0.3)	7/9(1.3 ± 0.1)	3/9(<1^c^)	6 ± 0.4	10 ± 0.2
	Airborne contact^a^	5/9(3.5 ± 0.1)	7/9(2.5 ± 0.3)	2/9(<1^c^)	2/9(<1^c^)	0/9	3/9(1.6 ± 0.2)	0/9	0/9	7 ± 1.5	9 ± 2.9
VF47	Directly inoculation^a^	9/9(4.2 ± 0.3)	9/9(2.5 ± 0.3)	0/9	0/9	0/9	2/3(2.45 ± 0.3)	7/9(1.5 ± 0.1)	2/9(1.15 ± 0. 2)	7 ± 0.2	10 ± 0.6
	Airborne contact^a^	5/9(2.2 ± 0.4)	7/9(1.2 ± 0.1)	6/9(<1^c^)	0/9	0/9	3/9(1.9 ± 0.1)	7/9(1.1 ± 0.5)	3/9(<1^c^)	7 ± 2.6	10 ± 1.1
nF52	Directly inoculation^a^	9/9(3.8 ± 0.2)	9/9(4.1 ± 0.2)	6/9(2.6 ± 0.1)	0/9	0/9	4/9(2.9 ± 0.3)	6/9(2.2 ± 0.3)	3/9(<1^c^)	7 ± 0.8	10 ± 1.7
	Airborne contact^a^	0/9	0/9	0/9	0/9	0/9	0/9	0/9	0/9	0	0

^a^number of positive tracheal or cloacal/total number of chickens

^b^Virus titers in tracheal or cloacal swabs are expressed as mean log10 EID_50_/ml ± SD

^c^Lower than the detection limit of 10 EID_50_

### Immunogenic drift of the passaged viruses

The immunization and challenge procedure used here is described in Table 3. These results show that all chickens immunized with PBS shed the indicated virus at 3 or 5 dpi (Table 3). In chickens immunized with F/98, those challenged with F/98 or nF47 were observed to shed less from the trachea and cloaca, respectively (Table 3). Conversely, chickens challenged with vF47 or with nF52 were found to shed more (Table 3). However, in groups immunized with one of the passaged viruses (nF47, vF47, nF52), less shedding was detected in the trachea and cloaca of chickens challenged with F/98 or their respective homolog viruses (Table 3).

**Table 3:** Isolation of H9N2 viruses in vaccinated chickens after challenged with the passaged viruses

Group	Challenge virus	HI mean titer(2^n^)	3dpi		5dpi	
		(Homologous antigen 2^n^)	Tracheal swab^a^ (Virus titers ^b^)	Cloacal swab^a^ (Virus titers^b^)	Tracheal swab^a^ (Virus titers ^b^)	Cloacal swab^a^ (Virus titers ^b^)
A (None-immune)	BSG	0	0/6	0/6	0/6	0/6
	F/98	0	6/6 (3.2 ± 0.3)	1/6 (1.6 ± 0.1)	3/6 (1.8 ± 0.2)	0/6
	nF47	0	6/6 (5.2 ± 0.1)	6/6 (3.1 ± 0.3)	2/6 (3.2 ± 0.1)	2/6 (1.1 ± 0.1)
	vF47	0	6/6(4.1 ± 0.2)	3/6 (2.1 ± 0.1)	6/6 (1.5 ± 0.3)	2/6 (<1^C^)
	nF52	0	6/6 (3.6 ± 0.1)	2/6 (3.8 ± 0.2)	6/6 (4.1 ± 0.2)	2/6 (1.6 ± 0.3)
B (Immune F/98)	F/98	7	1/6 (1.2 ± 0.1)	0/6	0/6	0/6
	nF47	7	1/6(3.1 ± 0.1)	0/6	2/6 (1.3 ± 0.3)	0/6
	vF47	7.3	6/6 (4.3 ± 0.1)	4/6 (2.2 ± 0.2)	6/6 (2.7 ± 0.2)	5/6 (1.0 ± 0.2)
	nF52	7	6/6 (3.9 ± 0.3)	2/6 (1.6 ± 0.1)	6/6 (2.1 ± 0.1)	3/6 (<1^C^)
C (immune nF47)	F	7	0/6	0/6	0/6	0/6
	nF47	7	0/6	0/6	0/6	0/6
D (immune vF47)	F	6.7	0/6	0/6	0/6	0/6
	vF47	6.7	0/6	0/6	0/6	0/6
E (immune nF52)	F	7	1/6(1.9 ± 0.3)	0/6	0/6	0/6
	nF52	7	0/6	0/6	0/6	0/6

^a^number of positive tracheal or cloacal swabs /total number of chickens

^b^Virus titers in tracheal or cloacal swabs are expressed as mean log10 EID_50_/ml ± SD

^c^Lower than the detection limit of 10 EID_50_

## Discussion

Previous work studying the evolution of influenza focused on performing sequence analyses of field isolates [1, 6, 8, 11, 12, 16, 17, 22, 23, 25, 32]. However, given the extensive use of vaccines in both humans and animals, it is also important to investigate the role of vaccine-induced antibody activity on influenza virus evolution. To date, there have been few reports about influenza virus escape mutants; the reported mutants were selected by host SPA in MDCK cells, by monoclonal antibodies in mice [20, 21, 33], or by polyclonal antibodies in pigs [18, 34]. While those reports show that SPA plays an important role in the evolution of influenza virus [22], neither embryonated chicken eggs nor chickens have been used to explore the effect of SPA on the evolution of H9N2 avian influenza virus, despite chickens being one of the most important hosts of influenza virus. Herein, we developed a chicken egg model to characterize the evolution of H9N2 avian influenza virus F/98, the vaccine seed in China and to serially passage this virus in SPF embryonated chicken eggs with or without SPA. Three groups of mutations distributed in the passaged viruses nF47, vF47 and nF52 were found.

Group one, containing mutants S145 N, G181E, and A198V in HA, was found in both the vF47 and nF52 virus. The antigenicity of the vF47 and nF52 viruses were changed with 32-fold HI titers lower than the parental virus F/98. Those changes resulted in low levels of protection against the passaged viruses vF47 and nF52 in chickens that were immunized with the vaccine based on the parental virus F/98 (Tables 1 and 3). In comparison, the results of the HI titer of nF47 was only 2-fold lower than the parental virus F/98 and resulted in good protection against the nF50 in chickens immunized with parental virus F/98. This indicates that mutants S145 N, G181E, A198V in HA may contribute to the antigenic drift of the v47 and n52 virus. These mutants are close to the hemagglutinin receptor-binding site and are consistent with the fact that substitutions near the hemagglutinin receptor-binding site determine the antigenic evolution of influenza A H3N2 viruses [35]. Since the vF47 and nF52 were adaptive virus with or without SPA, selective pressure of the antibody seems not to play the main role on antigenic drift in the egg model.

Group two included K131R in HA, C228Y and K375R in PB2, which appeared in three viruses: nF47, nF52, and vF47. The EID_50_ of each of the passaged viruses (nF47, vF47, and nF52) was significantly higher than that of the parental virus F/98 (Table 1). The replication abilities of the passaged viruses nF47, vF47, and nF52 in the trachea of chickens were significantly higher compared to the parental strain F/98 (Fig. 3a). This implies that the mutants K131R in HA, C228Y and K375R in PB2 may contribute to the enhanced replication of the passaged viruses. This result is consistent with previous reports of viruses passaged in mice and swine [18, 30, 36–38].

Interestingly, group three, consisting of A459T in NA, T156A and R189K in PB1, R99K in NP, and A143T in NS1, only appeared in the nF52 virus. Compared to the vF47 virus, the nF52 has a 9.3-fold higher incidence of EID_50_ (Table 1), significantly higher replication ability in the trachea and lung of chickens (Fig. 3a-d), and specifically lost the characteristic of airborne transmission. These differences between the vF47 and nF52 may result from the mutants of group three, which were not present in the vF47 virus generated in SPA. In other words, SPA induced by homologous maternal antibodies limited the vF47 virus to possess the mutants of group three (A459T in NA, T156A and R189K in PB1, R99K in NP and A143T in NS1), implying that SPA may regulate the genetic variation and replication ability of H9N2 viruses in the egg model.

## Conclusions

In summary, our findings suggest that the antigenic drift of H9N2 influenza virus occurs during serial passaging with SPA or SPF embryonated chicken eggs. The virus produced by passaging in the presence of SPA has fewer amino acid substitutions and displays earlier antigenic drift than the virus produced by passaging in SPF embryonated chicken eggs. In contrast, the virus passaged in SPF embryonated chicken eggs developed more genetic changes. These mutations lead to the virus having enhanced replication and pathogenicity in SPF chickens and changed the feature of airborne spread compared to the SPA-passaged virus. The vaccine prepared from the parental virus did not provide protection against the passaged viruses, but the vaccines produced from the passaged viruses conferred complete protection against the parental virus. Our findings broaden the understanding of antigenic drift and the selection of vaccine seeds for H9N2 influenza viruses.

## Abbreviations

BSL: Biosafety level
dpi: Days post-inoculation
EID_50_: The 50% egg infectious dose
EMDT: Embryo means death time
F/98: A/Chicken/Shanghai/F/98 (H9N2)
FBS: Fetal bovine serum
h: Hour
HA: Haemagglutinin
HI: Haemagglutination inhibition
MDCK: Madin-Darby canine kidney
nF47: The 47nd generation without selective pressure from antibodies
nF52: The 52nd generation without selective pressure from antibodies
OIE: Office international des epizooties
PBS: Phosphate buffer solution
RDE: Receptor-destroying enzyme
RT-PCR: Reverse transcription- polymerase chain reaction
SNP: Single-nucleotide polymorphisms
SPA: Selective pressures from antibodies
SPF: specific pathogen-free
vF47: The 47th generation under selective pressure on antibodies
